# Stress Granules in Respiratory Infections Linking Host Defence to Pathogen Adaptation and Therapeutic Opportunities

**DOI:** 10.1111/jcmm.71457

**Published:** 2026-11-13

**Authors:** Seoyeon Jo, Jaewhan Kim, Chang‐Hwa Song

**Affiliations:** ^1^ Department of Medical Science, College of Medicine Chungnam National University Daejeon Republic of Korea; ^2^ Department of Microbiology, College of Medicine Chungnam National University Daejeon Republic of Korea; ^3^ Translational Immunology Institute Chungnam National University Daejeon Republic of Korea

**Keywords:** antibiotic tolerance, biomolecular condensates, host defence, host‐directed therapy, infection, pathogen, stress granules

## Abstract

Stress granules (SGs) are dynamic, membrane‐less ribonucleoprotein condensates that assemble via liquid–liquid phase separation in the cytoplasm when translation initiation is impaired. SG formation commonly accompanies translational repression and may subsequently redistribute selected RNAs and signalling proteins, thereby linking cellular stress to innate immunity. Many viruses have been reported to inhibit SG assembly or recruit SG‐associated proteins, whereas bacterial and mycobacterial infections can activate eIF2α phosphorylation and are associated with SG‐like responses. Respiratory pathogens exhibit diverse SG phenotypes: coronaviruses and influenza viruses generally limit canonical SG assembly, although individual SARS‐CoV‐2 proteins have been reported to exert opposing effects; respiratory syncytial virus replication correlates with G3BP1 and SG‐positive cells, but an SG‐specific mechanism remains unresolved; and *Mycobacterium tuberculosis* induces persistent G3BP1‐positive granules together with translational and metabolic suppression. Studies in bacterial model systems also suggest that pathogen‐intrinsic protein condensates may influence dormancy and antibiotic tolerance, but whether analogous condensates form during respiratory infection remains unknown. Understanding how respiratory pathogens alter the ISR, translation and SG‐associated pathways may reveal pathogen‐ and context‐specific therapeutic opportunities. This review summarizes mechanistic advances in coronavirus‐G3BP interactions, SG biology and pathogen‐specific observations; critically distinguishes causal evidence from correlation, as well as SG‐dependent from SG‐independent functions of commonly manipulated proteins; and discusses antibiotic tolerance and therapeutic hypotheses in light of their current evidentiary limitations.

## 1 Introduction

Respiratory diseases continue to be a major cause of morbidity and mortality worldwide. The coronavirus disease 2019 (COVID‐19) pandemic underscored the vulnerability of human populations to emerging respiratory pathogens and highlighted gaps in our understanding of host–pathogen interactions. Stress granules (SGs) represent one component of this interface. Initially characterized as transient assemblies of untranslated mRNAs under environmental stress, SGs are now considered dynamic hubs that may modulate translation, innate immunity and cell survival. Studies of viral and bacterial infections have reported associations between SG‐related responses and pathogen replication, inflammatory signalling and cell survival [1]. However, these observations do not invariably establish that SG assembly itself is causal because translational repression and multifunctional SG‐associated proteins may independently influence the same outcomes. Accordingly, SG‐directed host therapy should be considered a pathogen‐ and mechanism‐specific possibility rather than a general consequence of SG formation.

Viruses depend heavily on cap‐dependent translation for protein synthesis. Host stress sensors such as PKR and PERK can detect viral double‐stranded RNA or misfolded proteins and phosphorylate eIF2α, thereby suppressing translation [2]. The resulting accumulation of untranslated mRNAs and translation factors can promote SG condensation. In many settings, therefore, SG assembly initially occurs downstream of translational repression rather than causing it. Once assembled, SGs may nevertheless feed back on selected RNA and signalling pathways by concentrating or excluding specific molecules [2, 3]. SGs form in response to heat shock, oxidative stress, nutrient deprivation and infection, and contain translation initiation factors, RNA‐binding proteins and small ribosomal subunits [3]. Their functional effects should thus be separated experimentally from those of the upstream stress response.

Although the relationships among eIF2α phosphorylation, SG formation and antiviral responses have been reviewed extensively [1, 3, 4], several questions remain unresolved. First, individual SARS‐CoV‐2 proteins may either promote or inhibit SG formation depending on the experimental context, and specific residues mediate the interaction between G3BP1/G3BP2 and the viral nucleocapsid protein. Second, studies linking SGs to antimicrobial outcomes frequently manipulate the intergrated stress response (ISR) or multifunctional SG‐associated proteins rather than SG assembly selectively. Third, bacterial model systems can form pathogen‐intrinsic biomolecular condensates associated with dormancy and drug tolerance, but their relevance during respiratory infection remains to be demonstrated. This review therefore distinguishes observations of SG formation, evidence supporting functional involvement of an ISR‐G3BP‐SG axis and experiments that directly isolate SG assembly as a causal variable.

Figure 1 provides a conceptual overview of the events described in this review. The schematic representation illustrates a respiratory epithelial cell in which infection‐induced SGs are delineated as semi‐transparent, liquid‐like condensates that are enriched in mRNAs and RNA‐binding proteins. Respiratory RNA viruses are illustrated either interacting with SGs, representing proposed strategies to recruit SG‐associated components, or spatially separate from SGs, representing reported suppression or avoidance of canonical granules. Rod‐shaped bacteria are depicted at the cell surface and within phagosome‐like vesicles, with arrows indicating putative bacterial effectors that may modulate SG‐associated pathways. The figure provides a conceptual framework rather than a uniform causal model: diverse respiratory pathogens are associated with different condensate phenotypes, whose consequences for host antiviral and antibacterial immunity require pathogen‐specific experimental validation.

**FIGURE 1:**
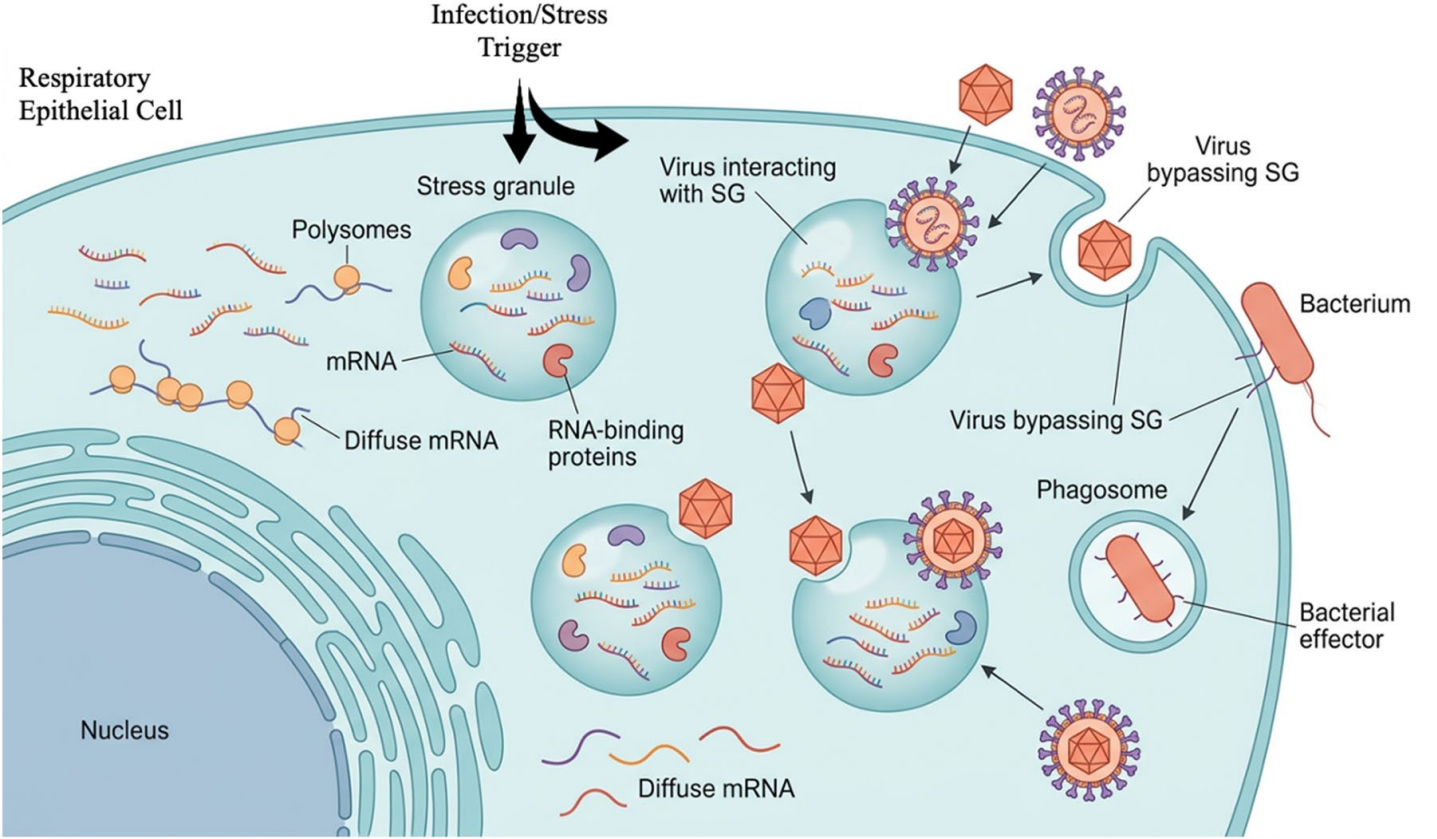
The formation of stress granules and the interactions between microbe and condensate in an infected respiratory epithelial cell. The schematic representation of a respiratory epithelial cell is shown, with the nucleus located on one side and the cytoplasm containing multiple stress granules (SGs) depicted as semi‐transparent, liquid‐like condensates. Within SGs, mRNAs (short coloured strands) and RNA‐binding proteins (various protein icons) are enriched, whereas additional mRNAs and polysomes remain dispersed in the surrounding cytoplasm. Respiratory RNA viruses are illustrated as non‐enveloped icosahedral particles and enveloped virions with surface spikes. Some virions interact with or become partially embedded in SGs (‘Virus interacting with SG’), while others remain separate (‘Virus bypassing SG’), representing distinct strategies to engage with or evade RNA‐protein condensates. Rod‐shaped bacteria are located at the cell surface and within a phagosome‐like vesicle (‘Bacterium’), with arrows indicating bacterial effectors (‘Bacterial effector’) that can modulate, target or circumvent SGs. Collectively, this figure conceptualizes the mechanisms by which infection‐induced SGs are formed in the respiratory epithelium and how diverse pathogens differentially interact with these membrane‐less condensates.

## 2 Stress Granules: Formation, Composition and Functions

### 2.1 Assembly Mechanisms

The formation of SGs occurs as a consequence of compromised translation initiation, most commonly through the process of phosphorylation of the α‐subunit of eukaryotic initiation factor 2 (eIF2α) at Ser‐51. The post‐translational modification in question has been shown to inhibit the guanine‐nucleotide exchange factor eIF2B, thus leading to the accumulation of translationally stalled 48S preinitiation complexes [5]. The condensation of these complexes with mRNAs and RNA‐binding proteins has been demonstrated to drive SG assembly through liquid–liquid phase separation (LLPS) [1]. Core SG proteins include G3BP1/2, T‐cell‐restricted intracellular antigen 1 (TIA‐1) and TIA‐1‐related protein (TIAR). The composition of SGs has been determined to consist of an internal core enriched in RNA–protein complexes and an outer shell with more dynamic constituents [1].

Although eIF2α phosphorylation is a major trigger for SGs, cells can form SGs via eIF2α‐independent mechanisms. Four eIF2α kinases have been identified as sensors of distinct types of stress: PKR is activated by viral dsRNA [6]; GCN2 responds to amino‐acid starvation; PERK to endoplasmic‐reticulum stress; and HRI to haem deficiency and mitochondrial stress [6, 7]. eIF2α‐independent SG assembly has also been described for the DEAD‐box helicase DDX3X, which nucleates SGs through its own low‐complexity domains rather than through translational shut‐off, illustrating that phosphorylation of eIF2α is sufficient but not strictly necessary for condensate formation. Viruses and bacteria frequently interfere with these kinases in order to maintain translation or modify SG responses [6].

PKR itself undergoes condensation upon encountering cytosolic dsRNA, forming dsRNA‐induced foci (dRIFs) enriched in ADAR1, PACT and NLRP1 that act as nucleation platforms for PKR activation and subsequent SG assembly [8]. These dRIFs form before translational repression, suggesting that PKR condensation amplifies the sensitivity of innate immune detection [8]. PACT has been shown to fine‐tune this process by suppressing PKR scanning along dsRNA in a length‐dependent manner, thereby preventing aberrant activation by endogenous dsRNA while permitting robust responses to viral dsRNA [9].

### 2.2 Compositions and Organization

Recent advancements in high‐resolution proteomics and microscopy have revealed that SGs are not homogeneous droplets, but rather layered assemblies. A recent overview of SG architecture described an inner layer enriched in translation initiation factors (eIF3, eIF4B, eIF4F complex); a middle layer dominated by scaffold proteins G3BP1/2, TIA‐1 and TIAR; and an outer layer containing variable signalling proteins, metabolic enzymes and RNA processing factors [4]. SGs contain translation‐blocked mRNAs, initiation factors (e.g., eIF4E, eIF4G, eIF4A, eIF3, eIF2), phosphorylated eIF2α, RNA‐binding proteins (e.g., TIA‐1, HuR, FMRP), regulatory proteins such as G3BP1, DDX6 and poly(A)‐binding protein (PABP) and interferon‐stimulated gene products like PKR and RIG‐I [3].

Time‐resolved proteomic profiling has revealed that SG composition undergoes systematic transitions across the SG life cycle. Early SG proteins, characterized by high phase‐separation propensity and high intrinsically disordered region (IDR) content, drive the rapid formation of an initial SG platform; late proteins are subsequently recruited as discrete modules that functionalise SGs for broader stress responses [10]. Proteins involved in LLPS are characterized by the presence of IDRs with low‐complexity regions (LCRs), which facilitate multivalent weak interactions. The biophysical properties of these regions determine whether condensates remain liquid‐like and reversible or transition to gel‐like states [10].

The scaffolding protein G3BP1 is a central nucleator of SG assembly. G3BP1 regulates LLPS through its N‐terminal NTF2 domain, central IDR and C‐terminal RNA‐binding domain [11] and can be activated by free RNA competing with intramolecular autoinhibitory contacts [12]. G3BP1 expression has been associated with SG formation and altered replication of multiple RNA viruses, and viral proteins including the SARS‐CoV‐2 nucleocapsid and NSP1 target G3BP1 [13]. Although the overall overlap among four independent SARS‐CoV‐2 nucleocapsid interactome datasets was limited, G3BP1 and G3BP2 were consistently identified, suggesting a central role in the nucleocapsid interaction network [14]. Importantly, G3BP1/2 also regulate mRNA stability, translation and innate immune signalling outside microscopically detectable SGs. Consequently, phenotypes caused by G3BP1/2 depletion or overexpression cannot by themselves be attributed specifically to SG assembly. Their multifunctionality in RNA metabolism, SG nucleation, PKR recruitment and innate immune activation makes them pivotal—but experimentally confounded—nodes at the intersection of stress and immunity [15, 16].

### 2.3 The Roles of SGs in Homeostasis and Immunity

SGs have been reported to facilitate cellular adaptation to stress by containing non‐translating mRNPs and permitting selective translation of some stress‐responsive transcripts. Beyond translation control, SG‐associated proteins may concentrate pattern‐recognition receptors such as PKR and RIG‐I and modulate interferon signalling [17]. TRIM25 co‐condenses with G3BP1 in a dsRNA‐dependent manner, and this spatial association was accompanied by enhanced ubiquitination of RIG‐I and antiviral signalling in the reported system [18]. These findings support a signalling role for specific condensates but should not be generalized to all SGs or stress conditions. Gadd45β has been reported to bind G3BP1, promote SG assembly and enhance type I interferon responses during RNA virus infection [19]. Conversely, loss of SG nucleators leads to heightened interferon production and subsequent toxicity [20]. Because the manipulated proteins also have SG‐independent functions, these studies support context‐dependent involvement of SG‐associated assemblies rather than establishing a universal effect of SG formation.

TLR‐IKK signalling has been reported to inhibit SG assembly downstream of pathogen recognition: prior activation of NF‐κB signalling by TLR agonists reduced SG formation while inflammatory cytokine production continued [21]. This reciprocal association is consistent with crosstalk between SG‐associated pathways and innate immune signalling. SGs have also been observed alongside changes in regulated cell death pathways. Early granules may sequester selected pro‐apoptotic proteins, whereas persistent stress and altered condensate clearance correlate with cell injury [22]; whether SG assembly is the direct mediator depends on the experimental system.

### 2.4 Distinguishing SG‐Dependent From SG‐Independent Effects

A central limitation of the field is that many perturbations used to alter SGs are not specific to condensate assembly. G3BP1/2 regulate mRNA stability, translation and innate immune signalling outside SGs, whereas ISRIB directly activates eIF2B and relieves ISR‐dependent translational repression; reduced SG formation is a downstream consequence. Thus, changes in pathogen replication after G3BP1/2 depletion, eIF2α‐pathway manipulation or ISRIB treatment cannot by themselves be assigned to SG assembly or disassembly.

Throughout this review, evidence is interpreted at four levels: direct causal evidence requires an SG‐selective perturbation or a separation‐of‐function rescue; supportive evidence uses convergent genetic and pharmacological approaches but retains plausible SG‐independent explanations; correlative evidence links SG abundance to a phenotype without isolating SG function; and inferred evidence identifies upstream ISR activation without directly demonstrating SGs. In many infections, translational repression precedes SG formation, making SGs initially a consequence or marker of stress. Once assembled, however, SGs may feed back on selected RNAs and signalling proteins. SGs are therefore treated here as context‐dependent potential modulators rather than universally causal structures.

### 2.5 The Disassembly of SGs and Granulophagy

The dissolution of SGs upon stress resolution is as important as their assembly. The clearance of SGs is facilitated by two principal mechanisms: disaggregation by molecular chaperones and selective autophagy, otherwise known as granulophagy [23]. Hsp70 chaperones localize to SGs and promote their clearance by preventing the accumulation of misfolded proteins. The AAA+ ATPase VCP/p97 has been shown to drive SG disassembly by unfolding and extracting ubiquitinated components from condensates [23]. Selective autophagy of SGs involves the receptor NS1‐BP, which interacts with GABARAP subfamily proteins and p62/SQSTM1; NS1‐BP loss has been shown to impair SG clearance and increase p62 ubiquitination [24]. Persistent SGs have been linked to chronic inflammation and may contribute to a cellular environment that supports long‐term pathogen survival. This concept is revisited in Section 4.1 in relation to *Mycobacterium tuberculosis* (Mtb) persistence, where sustained infection‐induced SGs are associated with prolonged translational and metabolic suppression in host cells, conditions that may favour bacillary survival.

### 2.6 Interactions Between P‐Bodies and RNA Processing

Stress granules have been observed to interact with processing bodies (P‐bodies), which are cytoplasmic condensates that are enriched in mRNA decay enzymes. Live‐cell imaging has demonstrated that SGs and P‐bodies frequently dock to one another, and that non‐translating mRNAs are capable of shuttling between these granules [25]. The phenomenon of viruses frequently appropriating both SGs and P‐bodies is well documented. For instance, adenovirus protein E4 11k has been observed to relocate P‐body proteins to aggresomes. Furthermore, West Nile virus and dengue virus recruit P‐body components, such as DDX6, to replication complexes. In addition, poliovirus and croxsackievirus have been demonstrated to degrade P‐body proteins [12].

## 3 The Role of SGs in Viral Respiratory Infections

Respiratory viruses are associated with highly pathogen‐specific SG phenotypes. Reported patterns include suppression of canonical SG assembly, recruitment of individual SG‐associated proteins to viral compartments or persistence of SGs in conditions that favour replication [26]. These categories are descriptive rather than uniformly causal, because viral replication can also be altered by upstream translational arrest or by SG‐independent functions of the same RNA‐binding proteins. Biomolecular condensates—including viral replication compartments and inclusion bodies—represent a recurring organizational principle in virus biology [27].

### 3.1 Coronavirus Infections (SARS‐CoV‐2 and HCoV‐OC43)

SARS‐CoV‐2 and the related seasonal coronavirus OC43 have been reported to limit canonical SG formation. In infected cell cultures, SGs were rare until approximately 24 h post‐infection, with fewer than 5% of cells displaying SGs despite ongoing viral replication [13]. These coronaviruses also reduced oxidative‐stress‐induced eIF2α phosphorylation and sodium‐arsenite‐triggered SG assembly in the tested systems [13]. The nucleocapsid protein (N) binds G3BP1 and has been reported to inhibit both phospho‐eIF2α‐dependent and ‐independent SG formation, whereas non‐structural protein 1 (Nsp1) suppresses eIF2α phosphorylation and reduces cellular G3BP1 [13]. G3BP1 overexpression was associated with reduced OC43 replication, supporting an antiviral function of G3BP1; however, this experiment does not establish that the effect was mediated specifically by SG assembly.

The SARS‐CoV‐2 N protein has been reported to impede canonical SG formation while remodelling SG‐associated components into atypical foci [28]. Because these foci lack some canonical SG markers, their effects on antiviral signalling should not be equated automatically with those of canonical SGs. The spike (S) protein has also been linked to changes in ADAR1‐p150 expression, dsRNA editing, PKR activation and SG abundance [29]. Variant‐comparison studies reported more SG‐positive cells after Omicron than after Delta or ancestral viral infection [30], but these observations do not by themselves identify SG as a determinant of lineage‐specific fitness.

Recent mechanistic studies refine the view that SARS‐CoV‐2 uniformly suppresses SG assembly. Structural and mutational analyses indicate that N engages G3BP1/2 through its N‐terminal ITFG motif (residues 15–18), with F17 contributing to binding of the NTF2‐like domain. G3BP residues V11, F15, Q18, F33 and F124 contribute to the binding pocket. Disrupting this interface impaired N‐mediated inhibition of SG assembly, providing relatively direct evidence that N‐G3BP binding contributes to the SG phenotype. Simultaneous depletion of G3BP1 and G3BP2 enhanced viral replication in A549 cells, whereas single‐paralogue depletion had limited effects. This supports redundant antiviral activity of G3BP proteins but does not determine how much of that activity requires canonical SG assembly rather than their SG‐independent RNA‐metabolic or signalling functions.

In contrast to the prevailing model of viral SG suppression, SARS‐CoV‐2 Nsp14 has been reported to promote SG formation through an N7‐methyltransferase‐dependent mechanism. In that study, Nsp14 increased *m* ^*7*^ *G* modification of host RNAs and their recruitment to granules through QK16 and QK17, and N antagonized this phenotype [14]. Thus, the net SG phenotype during infection may reflect opposing activities of viral proteins. ISRIB treatment reduced Nsp14‐dependent viral replication, whereas folic‐acid supplementation increased SG formation and viral titres [31]. Because ISRIB directly activates eIF2B and relieves ISR‐dependent translational repression rather than targeting SGs themselves, these findings cannot distinguish an SG‐dependent effect from broader changes in translation and stress signalling.

Evolutionary studies provide an additional layer of complexity. A truncated N protein, NM210, generated through the acquisition of a transcription‐regulating sequence during SARS‐CoV‐2 evolution, acts as a dsRNA‐binding protein that suppresses interferon responses and blocks G3BP1‐positive granules, including SGs and RNase L‐dependent bodies. Increased production of this truncated protein enhanced viral fitness in primary human cells and in mice. These findings provide a possible mechanistic basis for lineage‐dependent differences in SG induction, although direct evidence linking N expression to the reported differences between Omicron and earlier lineages should be stated cautiously [30, 32].

Residue‐level characterization of the N‐G3BP1 interaction provides a potential starting point for therapeutic development. A miniaturized 384‐well time‐resolved FRET assay has been established to identify compounds that disrupt this protein–protein interaction [33]. This platform enables screening, but whether candidate compounds restore antiviral activity through SG assembly, SG‐independent G3BP functions or both will require direct testing.

### 3.2 Influenza A Virus

Influenza A virus (IAV) depends on host cap‐dependent translation and is sensitive to conditions that induce early translational arrest. Pharmacological agents or oxidative stress that produce both translation inhibition and SG formation early in infection has been associated with reduced viral protein synthesis and replication [34]. IAV proteins NS1, NP and PA‐X antagonize PKR activation, SG assembly or host RNA expression [34]. These observations are consistent with selective pressure to preserve viral gene expression, but they do not isolate SG assembly from upstream translation repression as the antiviral mechanism. Accordingly, pharmacological enhancement of SG assembly should not yet be presented as an established influenza therapy; compounds would need to be tested under conditions that separate condensate formation from global translational inhibition.

### 3.3 Respiratory Syncytial Virus

RSV infection has been reported to induce SGs beginning approximately 12 h post‐infection and persisting during the viral life cycle [35]. SG‐positive cells contained more RSV protein, and G3BP1 knockdown reduced RSV replication [35], whereas viral RNA localized mainly to inclusion bodies and only transiently interacted with SGs. These findings show a proviral association between RSV replication, G3BP1 and SG‐positive cells. However, because G3BP1 has SG‐independent roles in RNA metabolism and signalling, they do not establish that canonical SG assembly itself facilitates RSV replication.

### 3.4 Other Respiratory Viruses and Paramyxoviruses

#### 3.4.1 MERS‐CoV

The infection of cells by MERS‐CoV does not result in the formation of stress granules [36]. The accessory protein p4a has been demonstrated to bind viral double‐stranded RNA and to prevent activation of PKR [36]. A recombinant MERS‐CoV lacking p4a still suppresses SG formation, indicating that additional viral antagonists operate during infection [36].

#### 3.4.2 Human Parainfluenza Virus Type 3 (HPIV3)

HPIV3 infection has been demonstrated to trigger SG formation through PKR‐mediated phosphorylation of eIF2α; viral mRNAs are sequestered into SGs and replication is inhibited [37]. As the infection progresses, N and P proteins form inclusion bodies (IBs) that suppress SGs by shielding viral RNA [37]. A comparable strategy has been documented for bovine parainfluenza virus type 3 (BPIV3), which has been shown to inhibit SG assembly and downregulate G3BP1 expression; the overexpression of G3BP1 has been demonstrated to restore SG formation and reduce viral replication [17].

#### 3.4.3 Measles Virus (MV)

MV infection has been demonstrated to induce SG formation in a PKR‐dependent manner [38]. ADAR1 deficiency has been shown to result in robust SG formation; however, this process can be counteracted through complementation with the p150 isoform of ADAR1, which has been demonstrated to reinstate SG inhibition and PKR deactivation [38].

#### 3.4.4 General Paramyxovirus Strategies

It has been established that a significant number of paramyxoviruses encode C proteins that limit viral dsRNA accumulation and inhibit SG formation [37]. Ebola virus has been observed to sequester SG proteins, including eIF4G, G3BP1 and TIA‐1, into viral inclusions [39]. West Nile virus and related flaviviruses have been shown to circumvent SG induction by maintaining low levels of viral RNA and membrane‐associated processes [40]. These varied strategies illustrate that viruses either induce then inhibit SGs, inhibit SGs outright or tolerate/exploit them [26]. Accumulation of copy‐back viral genomes (cbVGs) has been demonstrated to strongly activate PKR and induce SG formation [41]. In contrast, wild‐type Sendai virus produces minimal SGs due to its ability to express trailer RNAs containing a 5′ UUUUAAAUUUU motif. This motif binds the SG protein TIAR, thereby preventing SG assembly [42]. Table 1 summarizes the major respiratory viral pathogens, their stress granule responses and the associated host and viral mechanisms.

**TABLE 1:** Viral respiratory pathogens and stress granules.

Virus	SG response	Interpretation of host effect	Reported viral strategy
SARS‐CoV‐2/HCoV‐OC43	Canonical SGs are uncommon during infection (< 5% of cells in the cited study). N and Nsp1 inhibit SG‐associated responses, whereas Nsp14 has been reported to promote m7G‐dependent SG formation [13, 14, 30, 31].	G3BP1/2 exert redundant antiviral effects: simultaneous depletion increases viral replication. However, because G3BP1/2 also have SG‐independent functions, the contribution of SG assembly itself remains unresolved [13, 14].	N binds G3BP1/2 and remodels SG components into atypical foci; Nsp1 reduces eIF2α phosphorylation and G3BP1 abundance; Nsp14 promotes an opposing m7G‐dependent SG phenotype [13, 14, 28, 30, 31].
Influenza A virus	Early experimental conditions that cause translational arrest and SG formation are associated with reduced viral gene expression [34].	Reduced viral protein synthesis cannot be attributed solely to SGs, because global translation repression occurs upstream of SG assembly [34].	NS1 antagonizes PKR, whereas NP and PA‐X suppress SG‐associated responses [34].
Respiratory syncytial virus (RSV)	SGs appear approximately 12 h after infection and persist; viral RNA remains mainly in inclusion bodies [35].	SG‐positive cells contain more viral protein, and G3BP1 depletion reduces replication. These findings show an association but do not establish an SG‐dependent proviral mechanism [35].	RSV maintains SGs while segregating most viral RNA in inclusion bodies; the precise role of G3BP1 outside SGs remains unresolved [35].
MERS‐CoV	Infection does not efficiently induce canonical SGs in the reported model [36].	Continued translation is consistent with suppression of the PKR–eIF2α stress response, although an SG‐specific contribution was not isolated [36].	Accessory protein p4a sequesters dsRNA and limits PKR activation; additional viral factors may also suppress SG formation [36].
Human parainfluenza virus 3 (HPIV3)	PKR‐dependent SGs are induced early, whereas viral inclusion bodies later suppress SG formation [37].	SG appearance coincides with reduced replication, but PKR‐dependent translation arrest may contribute independently of SG assembly [37].	N/P‐protein inclusion bodies shield viral RNA and limit PKR activation and SG formation [37].
Measles virus	PKR‐dependent SGs are more prominent when the viral C protein is absent [38].	Loss of ADAR1‐p150 enhances PKR activation and SG formation; the relative contributions of translation arrest and SG assembly remain uncertain [38].	The C protein limits dsRNA accumulation, and ADAR1‐p150 suppresses PKR activation [38].
Ebola virus/West Nile virus	EBOV recruits SG‐associated proteins into viral inclusions, whereas WNV‐infected cells show limited spontaneous or arsenite‐induced SG formation [39, 40].	These observations indicate redistribution or avoidance of SG‐associated factors, but do not establish a uniform effect of canonical SG assembly on replication [39, 40].	EBOV sequesters SG‐associated proteins in inclusion bodies; WNV limits exposure of viral dsRNA and interferes with stress‐induced SG formation [39, 40].

### 3.5 DNA Viruses and Other Pathogens

Whilst the majority of respiratory pathogens are RNA viruses, it is evident that DNA viruses also modulate SGs. Kaposi's sarcoma‐associated herpesvirus (KSHV) has been shown to encode the lytic protein ORF57, which has been observed to bind the PKR‐activating protein PACT and PKR itself, thereby preventing their interaction and inhibiting PKR autophosphorylation [7]. The knockdown of PKR has been demonstrated to enhance the production of KSHV virions, thereby underscoring the antiviral function of the PKR‐eIF2α‐SG axis [7]. In the context of DNA virus infections in general, PKR can be activated by RNA polymerase III‐transcribed dsRNA species that are generated from viral DNA templates [9].

Adenovirus suppresses SG assembly during infection of epithelial cells: arsenite‐induced SGs are inhibited and P‐bodies relocate to the nuclear periphery. The deletion of the early gene E1A has been demonstrated to restore SG formation [43]. Adenovirus has been observed to reorganize RNA granules, thereby prioritizing the expression of late viral mRNAs and avoiding translational repression.

## 4 The Role of SGs in Bacterial Respiratory Diseases

Compared with the viral literature discussed in Section 3, evidence linking bacterial infection to SG biology remains limited. To avoid overstating the current state of knowledge, the following subsections distinguish between findings supported by direct experimental evidence of SG involvement and those that remain hypothesis‐generating. The latter include studies in which activation of an eIF2α kinase is inferred from exposure to a bacterial toxin or metabolite, without direct visualization or characterization of SGs during infection.

### 4.1 *Mycobacterium tuberculosis* Infection

Mtb infection has been reported to induce G3BP1‐positive granules in macrophages together with ISR activation and eIF2α phosphorylation [44]. Increased G3BP1 fluorescence and foci were also observed in infected mouse lung tissue and in bacterium‐containing bone‐marrow‐derived macrophages. The study reported localization of mTOR, Raptor and Ndufa12 to these granules, accompanied by reduced cap‐dependent translation, mitochondrial respiration and antimicrobial effector production [44]. ISRIB treatment restored translation, reduced the proportion of SG‐positive cells and lowered intracellular Mtb growth; G3bp1/G3bp2 double knockdown was likewise associated with preserved mTORC1 signalling and mitochondrial function [44]. These convergent interventions support functional involvement of an ISR‐G3BP‐SG axis in Mtb survival. Nevertheless, they do not isolate SG assembly as the sole causal mediator: ISRIB directly activates eIF2B and restores translation, whereas G3BP1/2 have SG‐independent roles in RNA metabolism, innate signalling and mTORC1 regulation. The findings should therefore be described as strong supportive evidence rather than definitive proof that SG assembly itself caused Mtb persistence. Rapamycin and everolimus inhibit mTORC1 and may affect tuberculosis through mechanisms such as autophagy; they should not be presented as restoring the same mTORC1 activity that was reduced in the study.

Mtb‐associated endolysosomal damage has also been linked to recruitment of G3BP1‐containing condensates [22, 44]. Such condensates can plug damaged endolysosomal membranes and facilitate repair [45], indicating a potentially host‐protective function that differs from the metabolic phenotype described above. Separately, the Mtb protein Mce3C has been reported to interact with lysosomal proteases and regulated cell death [22]. Taken together, these observations suggest that distinct stress‐induced G3BP assemblies may have different effects according to their subcellular location, timing and composition. They do not yet support a single model in which Mtb uniformly co‐opts SGs to suppress host defence.

### 4.2 *Pseudomonas aeruginosa* and Other Opportunistic Bacteria

*Pseudomonas aeruginosa* has been identified as a causative agent of hospital‐acquired pneumonia, with a particular prevalence among ventilated patients. Despite the absence of direct evidence for SG formation during *P. aeruginosa* infection, the pathogen activates eIF2α phosphorylation through iron depletion and the secretion of the redox‐active metabolite pyocyanin [46]. It has been established that other respiratory pathogens, including *Staphylococcus aureus* and *Klebsiella pneumoniae* , are capable of producing pore‐forming toxins. These toxins have been shown to activate the ISR, and it has been hypothesized that they are also capable of inducing SGs [46]. Because no study to date has directly visualized SGs in cells infected with any of these three organisms, the entries in this subsection should be read as candidate hypotheses that motivate future imaging and knockdown studies analogous to those already performed for Mtb (Section 4.1), rather than as established mechanisms.

### 4.3 Gram‐Negative Enteric Bacteria and Respiratory Infections

It has been observed that enteric pathogens, including *Salmonella enterica* serovar Typhimurium and *Shigella flexneri* , can, on occasion, result in respiratory disease in immunocompromised hosts. Studies conducted on fibroblasts have demonstrated that *Salmonella* induces transient SGs in approximately 5% of cells by 5 h post‐infection [46], whereas *Shigella* has been observed to elicit SGs in 20%–30% of cells [46]. The findings of this study suggest that *Shigella* can reduce drug‐induced SGs, indicating that it possesses effectors capable of inhibiting SG assembly [46]. The eIF2α kinases GCN2 and HRI have been identified as the mediators of this response, and the knockdown of both kinases has been shown to result in the complete abolition of SG formation [46]. Table 2 summarizes the reported and predicted stress granule responses of bacterial respiratory pathogens and their proposed mechanisms.

**TABLE 2:** Bacterial respiratory pathogens and stress granules.

Pathogen	Evidentiary status	SG response	Host effect/bacterial mechanism
*Mycobacterium tuberculosis*	Strong supportive evidence (in vitro and in vivo) but ISRIB and G3BP1/2 depletion are not SG‐specific [44]	Robust SG formation via eIF2α phosphorylation; sequesters mTORC1 (mTOR, Raptor) and mitochondrial complex I subunit Ndufa12 [44]	ISRIB and G3BP1/2 double knockdown restore translation and metabolism; reduces bacterial burden in vivo [44]
*Pseudomonas aeruginosa*	Hypothesis‐generating (indirect) [46]	Indirect evidence via pyocyanin activating eIF2α phosphorylation [46]	Potential SG formation; functional impact unconfirmed
*S. aureus/K. pneumoniae*	Hypothesis‐generating [46]	Pore‐forming toxins activate GCN2 and likely induce SGs [46]	Predicted to halt translation; host consequences unknown
*Salmonella typhimurium*	Established in vitro (fibroblasts) [46]	Transient SGs in ~5% of cells by 5 h; eIF2α phosphorylation via GCN2/HRI [46]	Early host stress response; effect on clearance untested
*Shigella flexneri*	Established in vitro (fibroblasts) [46]	SGs in 20%–30% of cells; reduces drug‐induced SGs [46]	Dual induction and inhibition of SGs; unknown effectors impede SG assembly

### 4.4 Other Respiratory Pathogens: Atypical Bacteria and Fungi

As previously mentioned, major viral and bacterial agents have been discussed. However, there are several other respiratory pathogens that have been shown to either trigger the ISR or alter eIF2α phosphorylation in host cells.

*Streptococcus pneumoniae* is the leading cause of community‐acquired pneumonia and releases hydrogen peroxide as a by‐product of metabolism. The induction of endoplasmic‐reticulum stress by this extracellular H_2_O_2_ is known to activate the PERK branch of the ISR [46]. Despite the absence of direct evidence for SG formation during pneumococcal infection, the observation of PERK‐dependent eIF2α phosphorylation suggests a potential for transient suppression of host translation and the subsequent assembly of SGs.

It has been established that *Staphylococcus aureus* and *Streptococcus pyogenes* are responsible for the production of pore‐forming toxins (α‐toxin and streptolysin O, respectively), which in turn activate the eIF2α kinases GCN2 and PKR [46]. The activation of these kinases is hypothesized to be the trigger for SG formation.

*Mycoplasma hyopneumoniae* has been observed to reduce host p‐eIF2α levels 24–36 h post‐infection [46], suggesting that mycoplasmas may actively reverse the ISR to maintain host translation and prevent SG formation.

*Histoplasma capsulatum* is a dimorphic fungus that is the causative agent of respiratory histoplasmosis. Furthermore, the infection of macrophages has been shown to increase p‐eIF2α levels at 12 h post‐infection, accompanied by activation of ATF4 and CHOP [46], implying that SGs may assemble during fungal infection. Table 3 summarizes pathogen‐specific eIF2α‐dependent stress responses and the evidence supporting potential stress granule formation.

**TABLE 3:** Pathogen‐specific eIF2α‐dependent stress responses and potential SG formation.

Pathogen	Evidence for SG involvement	Notes
*Streptococcus pneumoniae*	Activates PERK via H_2_O_2_; eIF2α phosphorylation [46]	SG formation inferred but not demonstrated
*Staphylococcus aureus*	α‐toxin activates GCN2 and PKR [46]	Predicted to induce SGs; functional impact unknown
*Streptococcus pyogenes*	Streptolysin O activates GCN2 [46]	Likely triggers SGs; host response unstudied
*Mycoplasma hyopneumoniae*	Reduces p‐eIF2α at 24–36 hpi [46]	May actively block SG formation
*Histoplasma capsulatum*	Increases p‐eIF2α; activates ATF4/CHOP [46]	Suggests SG assembly during fungal infection

## 5 Stress Granules, Cell Death and Bacterial Virulence Factors

In addition to the modulation of translation and innate immunity, studies have demonstrated that SGs intersect with programmed cell death pathways. The intracellular bacterium *Legionella pneumophila* secretes the effector kinase LegK3, which has been demonstrated to block activation of host caspases 3 and 7 and thereby prevent apoptosis [22]. *Mycobacterium tuberculosis* has been observed to induce lysosomal membrane damage in infected macrophages, leading to the formation of SGs and subsequent necrotic cell death. The secreted protein Mce3C has been shown to target lysosomal proteases, thereby blocking apoptosis and promoting necroptosis [22].

The bidirectional relationship between SGs and regulated cell death (RCD) pathways has been elaborated in recent work. The ability of SGs to inhibit apoptosis and pyroptosis early in infection is well documented, with the sequestering of key factors being a key mechanism in this process. In turn, damage‐associated molecular patterns (DAMPs) released during cell death have been shown to promote SG assembly, creating a feedback loop [22]. PANoptosis, defined as the co‐activation of pyroptosis, apoptosis and necroptosis, has gained significant traction in the scientific community as a response to respiratory pathogens. The relationship between SG signalling and PANoptosome activation represents a promising area for future investigation [22].

## 6 Host Stress Granules and Antibiotic‐Related Cellular Stress

This section considers whether host SG‐associated responses could modify antimicrobial outcomes. It does not treat pathogen‐intrinsic bacterial condensates as established components of host‐pathogen SG biology; evidence for those structures derives mainly from bacterial model systems. And is discussed as a future research direction in Section 7.4.

### 6.1 Current Evidence and Limitations

Antibiotic treatment can impose metabolic and oxidative stress on pathogens and host cells. Host SG formation during therapy could, in principle, alter translation and stress signalling, but direct evidence in respiratory infection models is lacking. Puromycin disrupts SGs by releasing nascent chains, anisomycin can increase eIF2α phosphorylation, and neomycin has been reported to reduce oxidative‐stress‐induced SG assembly [46]. These pharmacological observations demonstrate that antibiotics can alter stress pathways and granule abundance; they do not establish that host SGs determine antibiotic efficacy.

If SG‐associated translational and metabolic suppression reduces antimicrobial effector production during Mtb infection, it could in principle favour bacterial persistence. Conversely, preventing persistent SG assembly might enhance selected host responses. However, no study has established a direct causal relationship between host SG assembly and antibiotic tolerance or resistance in a respiratory infection model. Accordingly, the available evidence should be described as hypothesis‐generating and should not be used to infer that host SG modulation will uniformly improve antibiotic activity.

## 7 Context‐Dependent Therapeutic Implications and Future Directions

### 7.1 Pathogen‐Specific Antiviral Strategies

The available evidence does not support a universal strategy of promoting SG formation. For coronaviruses and influenza, conditions that induce translational arrest and SGs can reduce viral protein synthesis, but these effects may arise from global translation inhibition rather than from the condensates themselves. In other settings, including RSV and the reported Nsp14‐dependent SARS‐CoV‐2 pathway, SG‐associated phenotypes correlate with increased viral replication. Therapeutic direction should therefore be determined by the viral protein involved, infected cell type, infection stage and whether an intervention targets SG assembly, upstream stress pathway or a pathogen‐host protein interface. Indiscriminate SG induction could suppress essential translation or alter inflammatory phase separation; SG‐directed therapy remains an emerging therapeutic concept in respiratory disease, but pathogen‐specific validation is required [47].

### 7.2 Modulating the ISR‐Translation Axis in Bacterial Infection

In the Mtb study, ISRIB restored translation and mTORC1‐associated functions, reduced SG‐positive cells and lowered bacterial burden [44]. These results provide a preclinical rationale for investigating the ISR‐eIF2B axis as a host‐directed target, but ISRIB should be described as an eIF2B activator and ISR modulator rather than an SG‐specific inhibitor. Likewise, G3BP1/2 are not SG‐specific drug targets. Any intervention should determine whether efficacy depends on condensate assembly, translation recovery, metabolic reprogramming or innate signalling and should account for potentially protective G3BP condensates at damaged endolysosomal membranes. Rapamycin and everolimus act by inhibiting mTORC1 and should be considered separately as autophagy‐related host‐directed strategies rather than as agents that reproduce ISRIB‐mediated restoration of mTORC1 activity.

### 7.3 Targeting Defined Pathogen‐Host Interfaces

The mapped SARS‐CoV‐2 N‐G3BP1 interface offers a defined screening target, and a 384‐well TR‐FRET assay can identify compounds that disrupt this interaction [14, 33]. Such compounds would still require testing to determine whether antiviral effects arise through restoration of canonical SGs or other G3BP functions. Nsp14 is another antiviral target because of its essential enzymatic activities; however, its reported association with m^7^G‐dependent SG formation and viral replication does not justify assuming that global SG promotion or inhibition will be beneficial [31]. Similarly, blocking influenza NS1 or PA‐X, disrupting RSV‐G3BP interactions or enhancing TRIM25‐G3BP1 co‐condensation remain mechanism‐based hypotheses rather than validated broad‐spectrum SG therapies [18].

### 7.4 Future Direction: Pathogen‐Intrinsic Condensates, Dormancy and Antibiotic Tolerance

Studies in *E. coli* and other bacterial model systems have linked ATP‐sensitive protein condensates or aggresomes to dormancy depth, mRNA preservation and antibiotic survival [48, 49, 50, 51]. These bacterial structures are mechanistically distinct from eukaryotic host SGs. Whether Mtb, *P. aeruginosa. S. aureus* or *K. pneumoniae* form analogous condensates during respiratory infection, and whether such structures interact with host stress responses remains unknown. Future studies should test their formation in infection‐relevant models before incorporating them into therapeutic framework. This topic is therefore presented as a testable future direction, not as an established mechanism of host‐pathogen interaction.

### 7.5 Open Questions and Research Priorities

Several key questions remain unanswered. First, how does SG composition differ among respiratory epithelial cells, alveolar macrophages and dendritic cells? Second, which bacterial effectors directly induce or suppress canonical SG assembly? Third, can SG‐dependent effects be separated from upstream translational repression and from SG‐independent functions of G3BP1/2, PKR and other components? Fourth, do respiratory pathogens form pathogen‐intrinsic condensates during infection, as proposed in Section 7.4, and do these structures influence antibiotic tolerance? Fifth, how do time‐resolved compositional changes in SGs manifest during infection [10]? Finally, what is the context‐specific significance of G3BP‐containing condensates that plug damaged endolysosomal membranes [45]? Addressing these questions will require live‐cell imaging, proteomics, ribosome profiling, SG‐assembly‐deficient separation‐of‐function mutants, rescue experiments and infection‐relevant in vivo models.

## 8 Conclusions

Stress granules are dynamic consequences and potential modulators of cellular stress, translation and innate immunity. In respiratory infections, reported SG phenotypes vary by pathogen, cell type, viral or bacterial factor and stage of infection. SARS‐CoV‐2 and influenza A generally limit canonical SG formation, but SARS‐CoV‐2 proteins can exert opposing effects, and the contribution of SG assembly must be distinguished from G3BP1/2 functions and upstream translation control [14, 31, 33]. RSV replication is associated with SG‐positive cells and G3BP1, but an SG‐specific proviral mechanism has not been established. Mtb induces persistent G3BP1‐positive granules together with translational, metabolic and immune changes; ISRIB and G3BP1/2 depletion support involvement of an ISR‐G3BP‐SG axis, but neither intervention is specific enough to identify SG assembly as the sole causal mediator [44]. Accordingly, the available literature does not support a uniform therapeutic strategy of either increasing or decreasing SGs. More defensible therapeutic opportunities include targeting defined pathogen‐host interfaces or stress pathways in a pathogen‐, cell‐ and stage‐specific manner, while experimentally separating condensate effects from translation, metabolism and SG‐independent protein functions. Evidence connecting host SGs to antibiotic tolerance remains preliminary. Bacterial condensates associated with dormancy and antibiotic survival have been demonstrated mainly in model systems [48, 49, 50, 51], and their relevance to respiratory infection remains to be tested.

## Author Contributions

**Seoyeon Jo:** funding acquisition, writing – original draft, writing – review and editing. **Jaewhan Kim:** funding acquisition, writing – original draft, writing – review and editing. **Chang‐Hwa Song:** conceptualization, supervision, funding acquisition, project administration, writing – review and editing.

## Funding

This research was supported by the National Research Foundation of Korea (NRF) grant funded by the Korea government (MSIT) (RS‐2025‐02263643, RS‐2025‐00523465) and by BK21 FOUR Programme by Chungnam National University Research Grant, 2024 and 2026.

## Ethics Statement

The authors have nothing to report.

## Consent

The authors have nothing to report.

## Conflicts of Interest

The authors declare no conflicts of interest.

## Data Availability

Data sharing not applicable to this article as no datasets were generated or analysed during the current study.
